# Design parameters comparison of bubble column, airlift and stirred tank photobioreactors for microalgae production

**DOI:** 10.1007/s00449-023-02952-8

**Published:** 2024-01-16

**Authors:** Basar Uyar, Moussa Djibrine Ali, Gülsüm Ebru Ozer Uyar

**Affiliations:** 1 Department of Chemical Engineering, Kocaeli University, Kocaeli, Turkey; 2 Department of Plant Protection, Kocaeli University, Kocaeli, Turkey

**Keywords:** Microalgae, Photobioreactor, Characterization, *Chlorella sorokiniana*, Hydrodynamics

## Abstract

Microalgae are the most propitious feedstock for biofuel production due to their lipid and fatty acid content. Microalgae cultivation shares many features with bioreactors, such as thermal and pH regulation, feeding procedures, and mixing to enhance heat and mass transfers. Aeration and stirring speeds are important parameters to reduce the costs of producing microalgae. In this study, three different photobioreactor types (stirred tank, airlift, bubble column) were characterized and compared for microalgae production. Hydrodynamics, mass transfer, and power consumption were determined for various aeration rates (0.9, 1.2, 1.5 L/min), and stirring speeds (100, 200 rpm), and *Chlorella sorokiniana* growth performance was compared under the conditions that provided the highest volumetric mass transfer and the lowest mixing time. Photo-bioreactor homogenization was good as indicated by low mixing times (< 10 s). Bubble column had the highest volumetric mass transfer due to its sparger design. Gas holdup and volumetric mass transfer coefficient were found to increase with the air flow rate and stirring speed. For stirred tank, bubble column, and airlift photobioreactors, maximum specific growth rates of *C. sorokiniana* were 0.053, 0.061, 0.057 h^−1^, and biomass productivities were 0.064, 0.097, 0.072 gdw/L.day, respectively. Under the conditions tested, growth was limited by the volumetric mass transfer in the airlift and stirred tank and bubble column was the best option for producing microalgae. These findings pave way for more extensive use of these systems in producing microalgae and provide a basis to compare photobioreactors of different designs.

## Introduction

A bioreactor is a device or system that supports a biologically active environment under controlled conditions. A photobioreactor can be defined as a culture system in which light has to pass through the transparent reactor’s wall to reach the cultivated cells that carry out a light-dependent biological process [1].

Microalgae can be used as light-driven cell factories that convert CO_2_ to foods, feeds, high-value bioactives, and biofuels such as biodiesel derived from microalgal oil. This promising potential has created great interest in microalgal biotechnology as one of the emerging fields in the biotechnology era [2].

Different types of photobioreactors have been designed and developed for the production of microalgae. Those can be classified according to reactor geometry into flat panel, tubular, and vertical column photobioreactors.

Flat panel and tubular photobioreactors are commonly preferred because they show the highest efficiencies most probably due to their high illumination [3] but have their own challenges, such as temperature control, mixing, and aeration [4, 5].

Vertical column photobioreactors are mainly cylindrical vessels and can be divided into bubble columns, stirred tanks, and airlift reactors based on their mode of liquid flow and means of mixing.

Bubble column bioreactors (BCR) have the simplest design, a sparger attached at the bottom produces fine gas bubbles that provide mixing and aeration. It has the advantages of low capital cost, high surface area-to-volume ratio, lack of moving parts, satisfactory heat and mass transfer, relatively homogenous culture environment, and efficient CO_2_/O_2_ mass transfer. Airlift bioreactors (ALR) are vessels with two interconnecting zones. One of the tubes is called the riser where the gas mixture is sparged, whereas the other region is called the downcomer, which does not receive the gas. Generally, it exists in two forms: internal loop and external loop, depending on the downcomer configuration. Mixing is done by bubbling the gas through a sparger in the riser tube without any physical agitation. This decreases the density of the riser making the liquid to move upward. In the disengagement zone, the gas leaves the liquid, and the degassed liquid moves downwards in the annular space in a laminar fashion with defined and oriented motion. ALR has the characteristic advantage of creating a circular mixing pattern where liquid culture passes continuously through dark and light phases, giving a flashing light effect to algal cells. Stirred tank reactor (STR) is the most conventional design, where agitation is provided mechanically with the help of an impeller of different sizes and shapes. Air is bubbled at the bottom. A large disengagement zone separates the unused sparged gas and produced oxygen during photosynthesis from gassed liquid to the gas phase [6].

Vertical column photobioreactors are especially used on a laboratory scale; their main disadvantage is low surface area-to-volume ratio which in turn decreases light harvesting efficiency [6] therefore they are designed to have small radii to increase the surface–volume ratio [4] or require modifications to illuminate internal parts of the culture [7].

Bioreactor characterization is generally made for novel bioreactors to assess performance compared to existing systems or to compare different bioreactor systems. Characterization usually includes the determination of mixing time and liquid circulation time (liquid-phase characterization), gas holdup and bubble distribution (gas-phase characterization), microorganism growth rate and yield/productivity (biological characterization), volumetric mass transfer coefficient, and power consumption. The data obtained allow optimization of mixing, aeration, and mass transfer, and are also useful for modeling and scale-up studies.

The subject of this study is the comparison of three different photobioreactors in terms of hydrodynamic and mass transfer properties. It is also targeted to determine the most efficient and suitable photobioreactor type for microalgae production. In this scope, three photobioreactors with different operating mechanisms (STR, internal loop ALR, BCR) were characterized by means of mixing time and circulation time (liquid-phase characterization), gas holdup and bubble distribution (gas-phase characterization), microalgal growth rate and productivity (biological characterization), volumetric mass transfer coefficient, and power consumption. Attempts to compare two different photobioreactors for microalgae production are rare, and to the best of our knowledge, three photobioreactor comparison is nonexistent in open literature. This study intends to fill this gap, in anticipation of a more extensive use of these systems in producing microalgae.

## Materials and methods

### Photobioreactors

Photobioreactors used in this study are internal loop ALR, STR, and BCR.

All three had an inner diameter of 8 cm, an outer diameter of 8.4 cm, and a height of 48 cm, corresponding to a total volume of 2.4 L. The working volume of the photobioreactors was 2 L, and 0.4 L was left for headspace. This working volume corresponds to a height/diameter (aspect) ratio of 5.

The type of stirrer used in the STR was marine propeller with a diameter of 4.5 cm and a height of 1 cm. The clearance between the stirrer blade and the bottom of the STR was 8 cm. Aeration was provided by a single orifice aerator placed at the bottom center of the photobioreactor. Orifice’s inner diameter was 0.8 mm.

ALR was internal loop type, inner draft tube had a diameter of 5.8 cm and a height of 30 cm. The bottom clearance of the inner tube was 5 cm and the top clearance was 5 cm. Riser and downcomer cross-sectional areas were 26.4 and 23.8 cm^2^, respectively. Aeration was the same as STR, a 0.8 mm diameter single orifice aerator placed at the bottom center of the photobioreactor was used.

BCR had no inner parts, a microporous sparger (diameter of 7.2 cm) placed at the bottom was used for aeration.

The material of construction for all three photobioreactors was borosilicate glass (the draft tube in ALR was made up of transparent PVC), stirrer blade and shaft were made of stainless steel.

The air is supplied from the bottom of the photobioreactors by an air pump with a maximum capacity of 10 L/min, and air flow rate was controlled by a regulating valve and a rotameter. For tests of different aeration rates, air flow into each of the PBR was set to 0.9, 1.2, 1.5 L/min. The superficial velocity of air (U_G_) was calculated by dividing air flow rates to aerated cross-sectional area of PBRs (50.3 cm^2^ for STR and BCR, 26.4 cm^2^ for riser section of the ALR), which corresponded to U_G_ of 0.30, 0.40, 0.50 cm/s for STR and BCR, and 0.57, 0.76, 0.95 cm/s for ALR.

Each photobioreactor was equipped with a temperature probe that was inserted from the top of the photobioreactors and measured the temperature of the medium ~ 5 cm below the liquid surface. The probes were connected to a logging device, and the temperatures of photobioreactors and ambient air were recorded continuously during the bioprocesses.

**Fig. 1:**
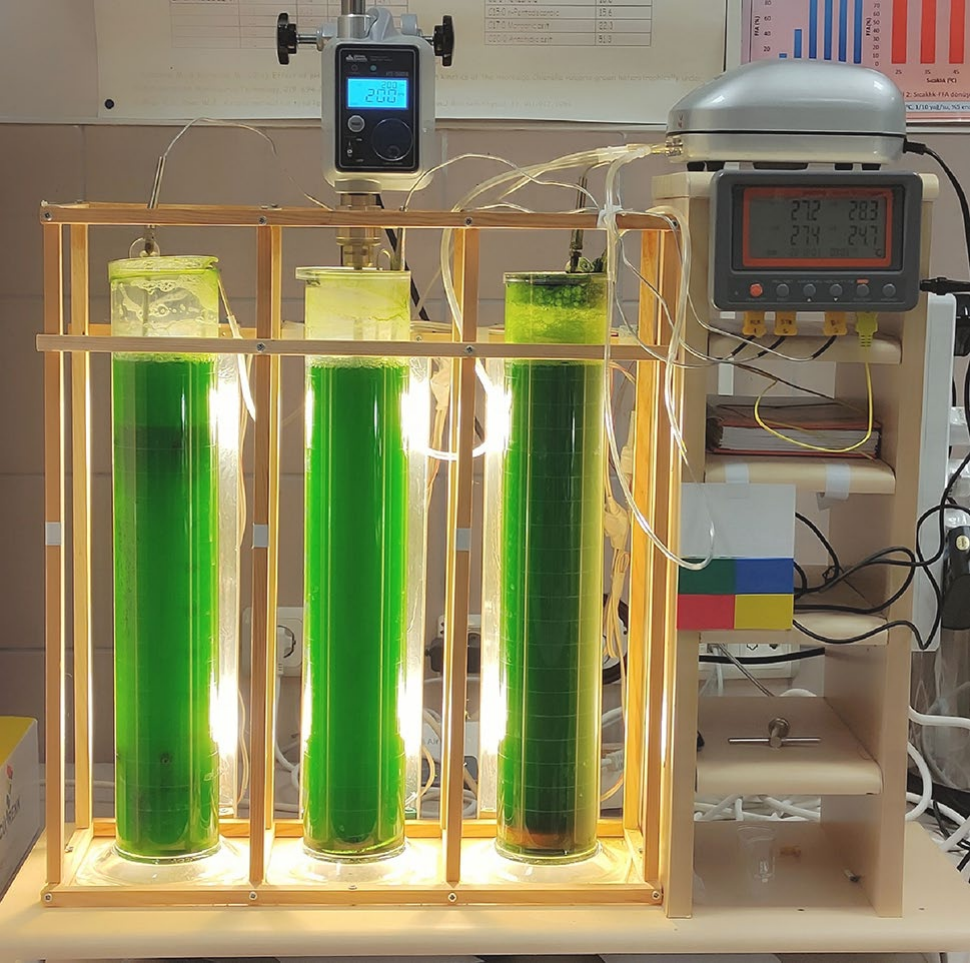
A photograph of the photobioreactor system during the runs. From left to right: ALR, STR (200 rpm), BCR

Figure 1 shows the photobioreactor system.

### Microorganism and growth conditions

Microalgae strain *Chlorella sorokiniana* was utilized in this study for the evaluation of the three photo-bioreactors.

Defined medium (BG11) was used for growing *C. sorokiniana*, and the composition was (g/L): NaNO_3_, 1.5; KH_2_PO_4_, 0.04; MgSO_4_.7H_2_O, 0.075; CaCl_2_.2H_2_O, 0.036; H_3_BO_3_ 0.003; Fe(III)citrate, 0.006; citric acid, 0.006 [8]. The initial pH of the solution was 7.8.

Characterization of hydrodynamic and mass transfer properties of photobioreactors was made in a biphasic (air–water) system, and a triphasic (microalgae-culture medium-air) system was used to determine bioprocess parameters. BG11 medium has low ionic strength (0.023 M) [9] and same density and viscosity as that of water [10]. Therefore, water was used as a substitute of culture media during tests.

One percent inoculation (20 mL to 2 L) of freshly grown microalgae was made into the photo-bioreactors.

The photo-bioreactors were operated in batch mode. Data reported are the average of two runs.

The study was carried out in indoor conditions under continuous artificial illumination. Illumination was provided from one side of the photobioreactors by two 5W E14 2700 K LED lamps per photobioreactor to obtain an average surface light intensity of 3500 lx. Light intensity measurements were made by a light meter (Extech Instruments).

### Characterization of the liquid phase

Characterization of the liquid phase was accomplished through mixing time, liquid circulation time, and liquid velocity.

The **mixing time** is the time required for the bioreactor composition to achieve a desired or specified level of homogeneity in the bioreactor. Mixing time is determined by introducing a tracer into the bioreactor at a single point and measuring the time tracer takes to propagate all of the bioreactor homogeneously. Full homogenization of the bioreactor takes a long time and therefore 95% homogenization is usually used to determine the endpoint. Common tracer compounds and sensors to measure tracer distribution are strong acid/alkali compounds and pH probes [11–15], salt solutions and conductivity probes [16–18], dye compounds, and cameras [14, 19].

In this study, salt solution and conductivity meter were tested first. However, the mixing time in photo-bioreactors was all small, and the response time of the conductivity sensor was not fast enough for accurate determination of the mixing time. Next, dye tracer was tested. Here, dye solution was prepared by mixing water with a water-soluble blue food-grade dye. One milliliter of this dye was introduced to the photo-bioreactor from the top part, the process was recorded by a digital camera at 60 frames per second rate, and the video was then analyzed frame by frame by image processing software. Software allowed selection of the photo-bioreactor area in the pictures and calculation of average red, green, and blue (RGB) color values of those pixels. The quantitated RGB color information was plotted versus time. Complementary color of blue is orange, and orange is a mix of two parts red and one part green. Therefore, change in red or green color can be used to track blue dye propagation in the photo-bioreactor. In our case, red color values were used. Red color value of the photobioreactor image starts to decrease with the addition of the blue dye, the decrease is mostly linear but slows down toward the end. The time elapsed between the dye injection and 95% homogenization is calculated and reported as the mixing time. This method has a very fast response time compared to the salt solution and conductivity meter method, thus allowed precise determination of the mixing time.

The **circulation time** is the time between two successive crossings of a tracer particle, in the same direction, through a chosen plane [20]. This parameter is calculated only for ALR which has a circulation loop, it is not applicable to BCR nor STR.

It is measured using a classical tracer response technique. The tracer can be a solution which can be tracked by an electrode (saturated solution of NaCl and conductivity probe [16] or a strong acid and a pH probe [21]) or a particle that can be visually tracked [20].

In this study, circulation time was determined by introducing small spherical rubber particles (~ 2 mm diameter) which have the same density as the culture medium to the photo-bioreactor so that the particles have neutral buoyancy and no net movement in non-aerated ALR. When the aeration started, the movements of the particles were recorded by a digital camera at 60 frames per second. The resulting video was then analyzed frame by frame and the time solid particles take to complete one full circulation in the ALR was determined. The measurement was performed at least twenty times for each condition and the average value is reported as the liquid circulation time.

**Liquid velocity** (or liquid circulation velocity) was also determined only for ALR, it is not applicable to BCR nor STR. It is calculated by dividing the length of the circulation loop by the liquid circulation time. In our case, the total length of one full circulation loop in the ALR was measured as 74 cm.

### Characterization of the gas phase

Characterization of the gas phase includes determination of gas holdup, average bubble diameter, and bubble size distribution.

The **gas holdup** was determined by dividing the volume of the gas phase in a photobioreactor by the total volume of the photobioreactor (gas + liquid) [13, 17]:

1 $$\varepsilon = \frac{V_G }{{\left( {V_G + V_L } \right)}}$$

where ε is the gas hold-up, V_G_ and V_L_ are volume of the gas and the liquid phases, respectively.

In order to calculate the gas holdup values, the air pump was shut down and the liquid level of a photo-bioreactor was measured first, then airflow was resumed and the new liquid level was measured. The difference between the two levels allowed calculation of the gas holdup in the photo-bioreactors.

**Bubble size distribution** and **average bubble diameter** are determined by measuring the diameter of air bubbles in the photobioreactors by photographic method [22–25]. Here, photobioreactors were operated under the desired conditions and close-up pictures were taken with a high-speed camera (exposure time of 0.001 s). The images were then analyzed in an image processing software (Paint.NET, free to use) to determine air bubble diameters. In average, 100–120 bubbles were selected and measured for each condition tested to calculate the average diameter and size distribution of air bubbles inside the photo-bioreactors. For large bubbles, the shape deviated from spherical to irregular, in those cases, diameter of the sphere which has the same area of the irregular shape was calculated and equivalent spherical diameter was found. Finally, arithmetic average of the bubble diameters was calculated and reported as the average bubble diameter.

### Volumetric mass transfer coefficient determination

The well-known dynamic gassing-in and gassing-out methods were used to measure the volumetric mass transfer coefficient (k_L_a) using a dissolved oxygen (DO) electrode (Mettler Toledo).

The photobioreactor was deaerated until the DO concentration had declined to below 5% of air saturation. Then a preset flow of air was established, and the increase in DO concentration was monitored until the DO concentration reached almost 100% of the air saturation value, which took between 2 and 10 min depending on the photo-bioreactor type and process conditions.

The k_L_a was then calculated as the slope of the linear equation:

2 $$k_L a = \frac{{\ln \left[ {{{\left( {C^* - C_0 } \right)} / {C^* - C}}} \right]}}{{\left( {t - t_0 } \right)}}$$

where C* is the saturation concentration of DO, C_0_ is the initial concentration of DO at time t_0_, and C is the DO concentration at any time t [11, 21, 26].

Mass transfer coefficients of O_2_ and CO_2_ are close since the difference is only due to the difference between their molecular diffusion coefficients in the liquid medium [17]. More specifically, k_L_a(CO_2_) can be calculated by multiplying k_L_a(O_2_) by 0.91 [27, 28].

In STR, k_L_a was also estimated according to the following equation:

3 $$k_L a = 0.026\left( {P / V} \right)^{0.4} U_G^{0.5}$$

which is accurate within approximately 20–40%, under conditions of; pure water, volume up to 2600 L, 500 < P/V < 10,000 (W/m^3^) [29].

For BCR, correlations suggested in the literature to estimate mass transfer coefficient are usually of the type [30]:

4 $$k_L a = aU_G^n$$

where various values of a and n are reported depending on sparger type, U_G_ range, solution used, PBR diameter, and flow regime inside BCR. The correlation that fits best to the operating conditions of this study is proposed by Shah et al. (1982) as k_L_a = 1.174·U_G_^0.82^ (sintered plate sparger, 10–15 cm PBR diameter, 2.5–4.4 m PBR height, U_G_ = 0.3–8 cm/s) with an error range of 17% [31].

For ALR, Kracik et al. (2019) proposed the following correlation to estimate kLa under conditions of aqueous solutions, laboratory size equipment, power input up to 600 W/m, U_G_ up to 0.025 m/s, superficial liquid velocity up to 0.1 m/s, with a relative standard deviation of 26% [32]:

5 $$k_La = 0.0103\left( {P / V} \right)^{0.6} U_G^{0.52}$$

### Characterization of biological parameters

Widely used parameters to compare microbial growth are specific growth rate, cell doubling time, and cell productivity.

The following relationship was used to calculate the **specific growth rate** (µ) of microalgae:

6 $$\mu = \frac{{\left[ {\ln \left( {N_2 } \right) - \ln \left( {N_1 } \right)} \right]}}{t_2 - t_1 }$$

where N_1_ and N_2_ are cell biomass concentrations (g/L) at times (t_1_) and (t_2_) [33].

**Doubling time** of cell mass (t_d_) was then defined as:

7 $$t_d = \frac{\ln \left( 2 \right)}{\mu }$$

**Biomass productivity** (Px) is expressed in terms of volumetric productivity (g L^−1^ d^−1^). It was calculated using the following formula:

8 $$Px = \frac{{\left( {N_2 - N_1 } \right)}}{{\left( {t_2 - t_1 } \right)}}$$

### Volumetric power consumption

Volumetric power consumption in STR and BCR due to aeration was calculated using:

9 $${P / V} = \rho gU_G$$

where P is the power supply by aeration (W), V is the working volume of PBR (m^3^), ρ is the density of water (1000 kg/m^3^), g is the gravitational acceleration (m/s^2^), and U_G_ is.

the superficial gas velocity based on the total cross-sectional area of the PBR (m/s).

In case of ALR, the following equation was used since aeration is only provided in the riser section [13, 14, 16]:

10 $$\frac{P}{V} = \frac{\rho gU_G }{{\left( {1 + {{A_d } / {A_r }}} \right)}}$$

where A_d_ is the cross-sectional area of the downcomer zone (m^2^), and A_r_ is the cross-sectional area of the riser zone (m^2^).

In STR, additional volumetric power consumption due to stirrer was calculated using [34]:

11 $$\frac{P}{V} = \frac{{\left( {N_p \rho N^3 d^5 } \right)}}{V}$$

with N_p_ being the impeller power number, N the agitation speed (rps), d the impeller outer diameter (m).

For this study, V = 0.002 m^3^, U_G_ = 0.0030 to 0.0095 m/s, A_r_ = 0.00264 m^2^, A_d_ = 0.00238 m^2^, N_p_ = 0.38, N = 1.67 to 3.33 rps, d = 0.045 m.

### Analytical methods

Off-site analyses were made to monitor microalgal growth and pH in photobioreactors during the runs, and to determine lipid content, protein content, the fatty acid profile of microalgal biomass, and the sugar and organic acid content of culture media after the runs.

Photobioreactors were regularly sampled for pH and cell growth analysis. A pH meter (Mettler Toledo) was used to measure the pH of the samples.

Microalgal growth was monitored spectrophotometrically at a wavelength of 600 nm (Jenway 6800 UV–VIS spectrophotometer). For this particular microalgae strain (*C. sorokiniana*), an optical density of 1.0 at 600 nm was found to correlate to a biomass content of 0.15 gdw/L.

After the runs were finished, the photobioreactor contents were centrifuged at 4000 rpm for 10 min, the supernatant was discarded, the biomass was washed and centrifuged again and then dried in an oven at 60 °C until a constant weight was reached in order to measure the dry weight of the microalgae biomass. Dried biomass was also required for lipid content, fatty acid distribution, and protein analysis.

Lipid content analysis was made by solvent extraction. Protein content was estimated by the Kjeldahl method. Fatty acids of biomass were profiled by GC analysis, and the sugar and organic acid content of culture media were determined by HPLC.

## Results and discussion

### Mixing time

Mixing of microalgae cultures is necessary to prevent sedimentation of algal cells, ensure that all cells of the population have uniform average exposure to light, pH and nutrients, facilitate heat transfer, and improve the gas exchange between the culture medium and the air phase. Therefore, mixing can considerably enhance productivity for a wide range of operational conditions. Mixing time measurement is important because it is relevant to the length of time for which detectable inhomogeneities last in the extremities of the vessel and can identify the extent of possible concentration gradients in a photobioreactor [4, 12, 16, 19].

**Fig. 2:**
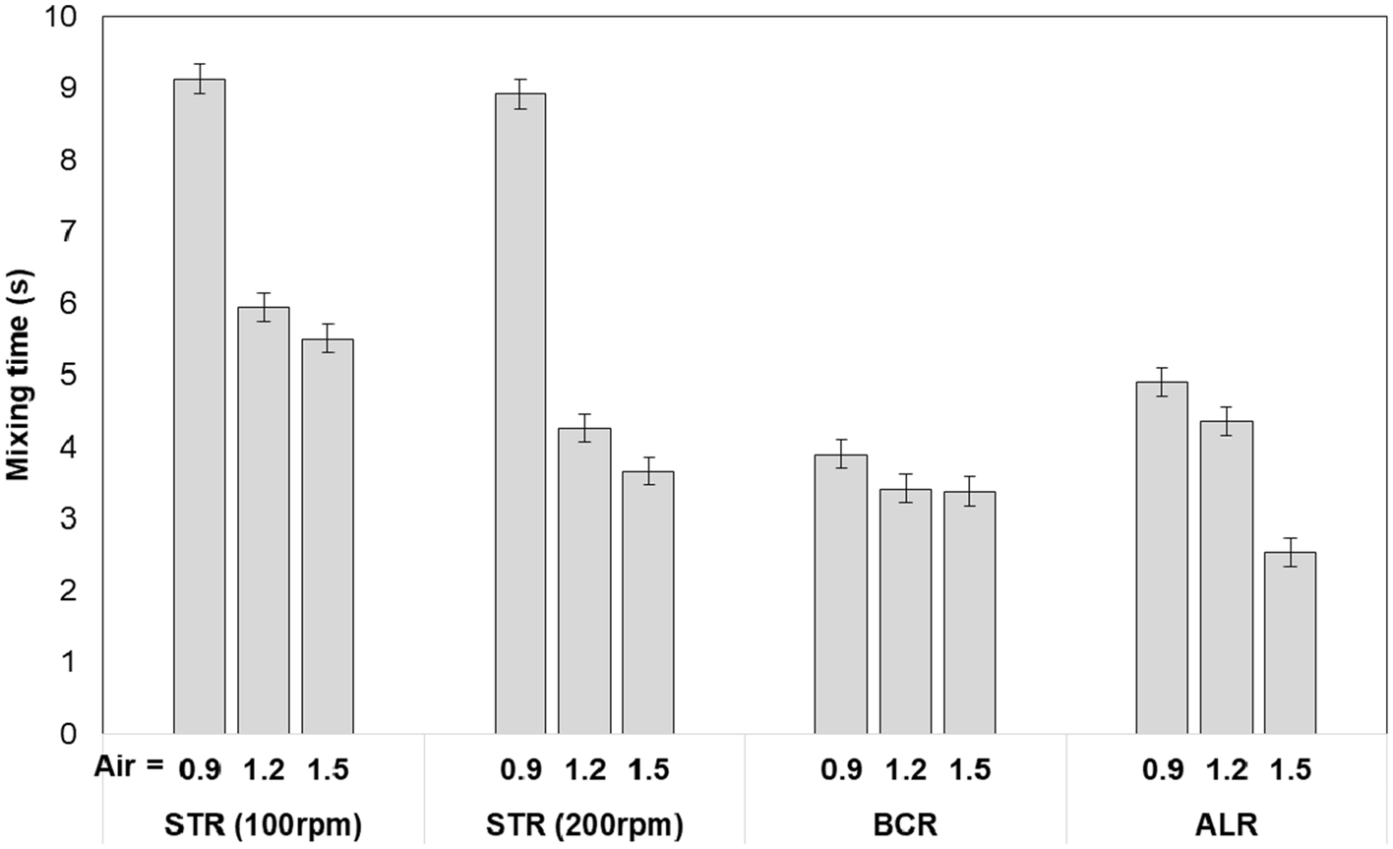
Effect of air flow rate (0.9, 1.2, 1.5 L/min), and stirring speed of STR (100 and 200 rpm) on mixing time of photobioreactors

In this study, the effect of air flow rate (0.9, 1.2, 1.5 L/min) and stirring speed (100 and 200 rpm, for STR only) on the mixing time of photobioreactors were determined and shown in Fig. 2.

Since the photobioreactors operated were all small lab scale, mixing times were low and homogenization was achieved in less than ten seconds for all cases tested.

Airflow into the photobioreactors causes mixing due to moving bubbles (also called pneumatic agitation) and therefore increasing airflow is expected to increase the mixing and decrease the mixing time [13–15]. In STR, increasing the stirring speed should provide better mixing and thus decrease the mixing time [12, 35].

Our results showed that in STR, the air flow into the photobioreactors was an effective factor that caused mixing: increasing air flow rate from 0.9 to 1.5 L/min) decreased the average mixing time from 9.03 to 4.59 s. Increasing the stirring speed also decreased the mixing times as expected, however the stirring speed had a minor effect on the mixing time compared to the air flow rate, increasing the stirrer speed from 100 to 200 rpm decreased the mixing time by 18% on average, all the cases considered. STRs are usually equipped with baffles to improve stirring efficiency, lack of baffles in this STR setup probably limited effect of stirring speed on the mixing time. It should also be noted that height-to-diameter ratio of STR used was also unusually high (H/D = 5) which also limited effect of stirrer speed on the mixing time. Using more stirrers equally spaced along the central shaft or decreasing height-to-diameter ratio would improve overall mixing. Indeed, decreasing H/D from 5 to 3 decreased average mixing time from 6.24 to 2.70, and at H/D = 3 increasing the stirrer speed from 100 to 200 rpm decreased the mixing time by 22% on average (results now shown).

In BCR, the effect of air flow rate on the mixing time could not be determined decisively from the data, observation of the tracer dye behavior in the BCR showed that without stirrer blades (STR) or draft tube (ALR) to direct the air bubbles, significantly more radial (lateral) movement of air bubbles were available and that caused back mixing, mixing loops, temporary dead zones, bubble merging, and splitting. McClure et al. (2015) reported a similar finding, they found that there was little change in the mixing time for different superficial air velocities in the range of 0.07–0.29 m/s [18].

In the case of ALR, increasing air flow rate from 0.9 to 1.2 L/min decreased mixing time by 11%, and increasing from 1.2 to 1.5 L/min decreased the mixing time by a further 42%. Our results support the findings of Guo et al. (2015), who found that the mixing time decreases with the increase of the gas rate in an ALR [16]. They suggested that the mixing time is primarily controlled by the liquid turbulence and cycling frequency in the ALR, both of which depends directly on the magnitude of the induced liquid circulation velocity. Therefore, the mixing time becomes shorter with the increase of the liquid circulation velocity. Banerjee et al. (2020) also found a similar result, increasing air flow rate from 0.1 L/min to 0.8 L/min decreased mixing time from 86 to 40 s in a 1.4 L single orifice ALR [27].

Overall, when all three photobioreactors are compared; for air flow rate of 1.5 L/min, ALR had the smallest mixing time (2.53 s compared to 3.38 for BCR and 3.66 for STR at 200 rpm), however for air flow rates of 0.9 and 1.2 L/min, BCR had the smallest mixing time (3.42 s compared to 4.35 for ALR and 4.26 for STR).

Therefore, it can be suggested that mixing in BCR was the most efficient, followed by ALR and then STR. The advantage of BCR here can be attributed to the type of sparger; in the BCR, micropore sparger was used, whereas in the ALR and STR, single-orifice tube sparger with an inner diameter of 0.80 mm was used.

Riet et. al. (2011) calculated mixing time values for STR, ALR, and BCR for different conditions (height-to-diameter ratio, bioreactor volume, gas velocity). At H/D of five, mixing times were reported to be in the order of BCR < ALR < STR, for all bioreactor volumes and gas velocities calculated, which confirms findings of this study. They concluded that when one considers mixing, BCRs are by far to be preferred in most cases because of lower mixing time, lower power consumption, simple mechanics and the reason to use STR might be their ability to handle viscous broths better and higher volumetric productivity [36].

A similar result was obtained by Miron et al. (2004), who claimed that for a given aeration velocity, the BCRs produced a shorter mixing time compared with the ALRs because compared with the chaotic flow in the BCRs, the organized cyclic flow in the ALRs impeded bulk mixing [15].

It should also be noted that the mixing time values for all were very low and mostly close to each other. Considering a measurement error margin of ± 0.2 s due to non-instantaneous injection of the tracer and moving air bubbles that affect the color detection, the conclusions that can be drawn here may be disputed by further and more accurate research.

Previous studies on the mixing time in photobioreactors reported a wide range of values, possibly due to different photobioreactor sizes and operating conditions. This difficulty was also acknowledged by Mc Clure et al. (2015) who stated that very different values are observed in the literature since the mixing time depends on the column geometry and size, the type of sparger, on the superficial gas velocity, but it is also strongly influenced by the injection and measurement locations [18].

In a study with very small-scale flat panel ALRs (15 mL), the mixing time showed an exponential decrease as the rate of aeration increased. For 0.1 to 1 L/h aeration, it decreased from 34.5 to 15 s, then from 1 to 5 L/h it further decreased from 10.5 to 1.5 s [37].

In another study, again very small-scale (15 mL) ALR and BCRs were compared, the mixing times were found to be almost the same for both photobioreactors, and they were reduced from 2.7 to 0.7 s while the airflow rate increased from 0.6 to 2.4 L/h. The author suggested that the diameter of the gas sparging disk had more effect on the mixing time compared to the photobioreactor geometry [19].

In a comparable sized STR (3L, H/D = 1.15) and air flow rates (0.33–1.67 L/min), Hadjiev et al. (2006) reported the mixing times between 6 and 26 s depending on the gas flow rate and the stirrer configuration and also showed that in a single phase (no air) system the mixing times were 20 to 95 s, proving the effect of aeration on the mixing [12]. In a similar setting (2.5L STR), mixing times of 2 to 15.5 s were found depending on the airflow rates (0.5 to 2 vvm) and the stirring speeds (10 to 800 rpm) [35].

In larger scales, higher mixing times were reported. For 60 L working volumes, the mixing time was decreased from 130 to 110 s for ALR and decreased from 120 to around 60 s in BCR as the air velocity increased from 0.003 to 0.05 m/s [15].

In a 50 L working volume photobioreactor, the mixing time was in the range of 6 to 44 s depending on the air velocity [14]. In a 150 L flat panel photobioreactor, the mixing times of 40 to 200 s were reported, again depending on the air velocity [13].

### Bubble size distribution, average bubble diameter, gas holdup

**Fig. 3:**
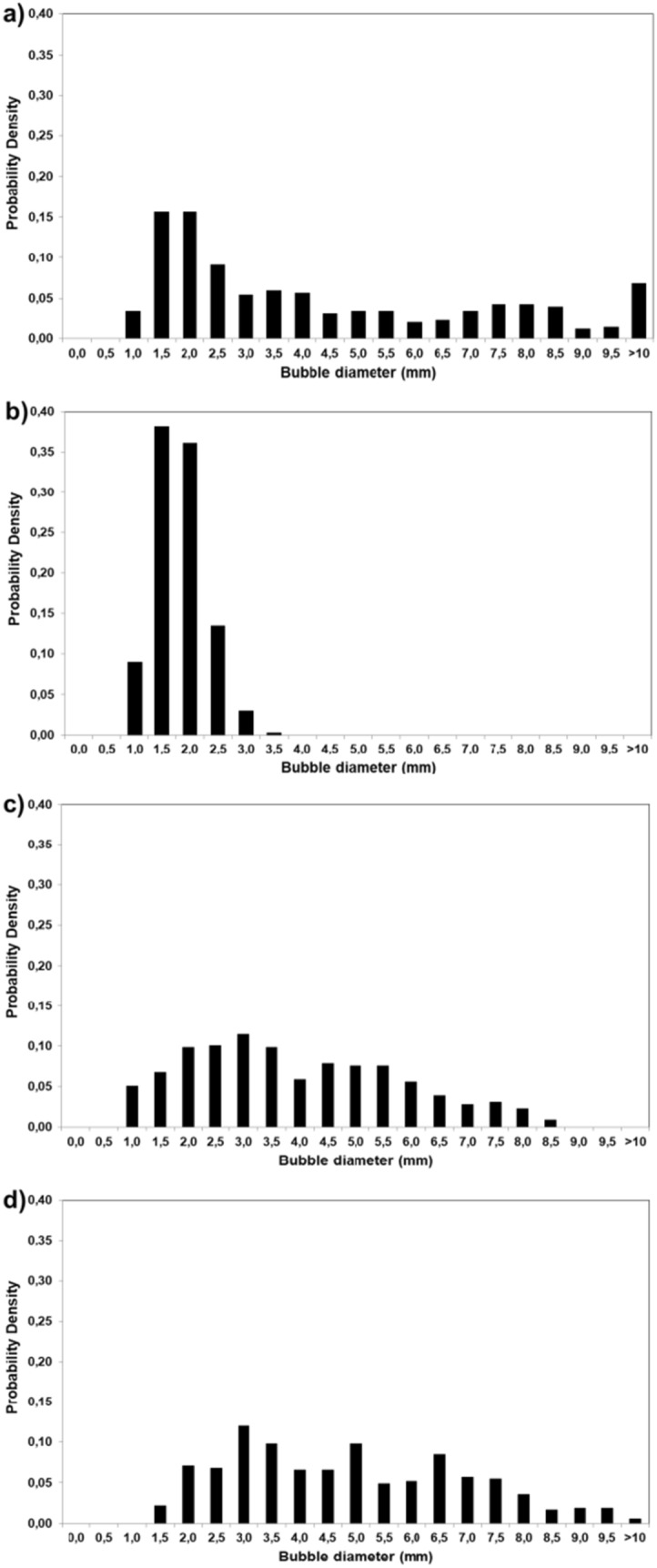
Bubble size distribution at aeration rate of 1.5 L/min in; (a) ALR, (b) BCR, (c) STR at 100 rpm, (d) STR at 200 rpm

Figure 3(a–d) represents the distribution of bubble sizes in the three photobioreactors that operated under the same aeration rate (1.5 L/min). The difference in BCR compared to STR and ALR is obvious. Microsparger in BCR produced smaller bubbles and also, a much narrower size distribution was observed. The distribution in the ALR was also different from STR, although both have the same type of sparger and comparable average bubble diameters; the distribution in ALR was bimodal, whereas in STR, unimodal no-normal distribution was obtained. In STR, no-normal distribution of air bubbles was also reported previously [25].

The present study showed that the average sizes of bubbles in ALR and STR were almost the same (4.25 and 4.18 mm), and significantly larger than that in BC (1.56 mm). Clearly, the air sparger is the key determining factor in the average size of the bubbles. The same type of sparger was used in the ALR and the STR (single orifice with an inner diameter of 0.8 mm), however in BCR, microporous sparger was used which resulted in much smaller air bubbles.

In ALR, for U_G_ of 0.57, 0.76, 0.95 cm/s cm/s, average bubble diameters were determined to be 3.6, 4.1, 4.8 mm, and maximum bubble sizes observed were 8.9, 14.9, 16.3 mm, respectively, which clearly shows that bubble size increases with increasing air velocity.

Deng et al. (2010) investigated the influence of superficial gas velocity on the bubble diameters in an ALR and reported that the bubble diameter slightly increased with an increase in gas velocity, which supports our finding [38].

In BCR, average bubble diameters were found to be not dependent on the air velocity; average bubble diameters were 1.7, 1.5, and 1.6 mm for air flow rates of 0.9, 1.2, and 1.5 L/min, respectively.

This finding is in agreement with other works, where Sauter mean diameter of bubbles were measured for different air velocities in a BCR, and the average bubble diameters were again found to be independent of air velocity [39, 40].

In STR, at 100 rpm stirring speed, average bubble diameters were 4.3, 3.7, and 3.2 mm for air flow rates of 0.9, 1.2, and 1.5 L/min, respectively. At 200 rpm stirring speed, average bubble diameters were 5.6, 4.7, and 3.6 mm for air flow rates of 0.9, 1.2, and 1.5 L/min, respectively. These results show that the average bubble diameters were found to decrease as the air velocity increased. Although generally bubble diameter was found to decrease as the stirring speed increased [22, 41], the results of this study showed the contrary. There are conflicting findings from other studies; Ramezani et al. (2015) reported that the bubble diameter increased as the stirring speed and gas flow rate increased [23], whereas in another work, authors reported that the effect of airflow rate on the bubble diameter was very small and the stirring speed did not result in a measureable effect [24]. The different findings are possibly due to the added complexity of the stirrer and other factors, such as photo-bioreactor geometry, placement, and type of stirrer blade, which also play a non-negligible role. Xiao et al. (2020) who used a 43 L STR (H/D = 1) with 0.2 M NaCl solution also observed that the bubble sizes on the positions below the impeller increase slightly with the increase of the impeller speed, and attributed this to the increase in flow intensity which allows larger bubbles to be recirculated into the lower vortex region [41], which is another plausible explanation.

**Fig. 4:**
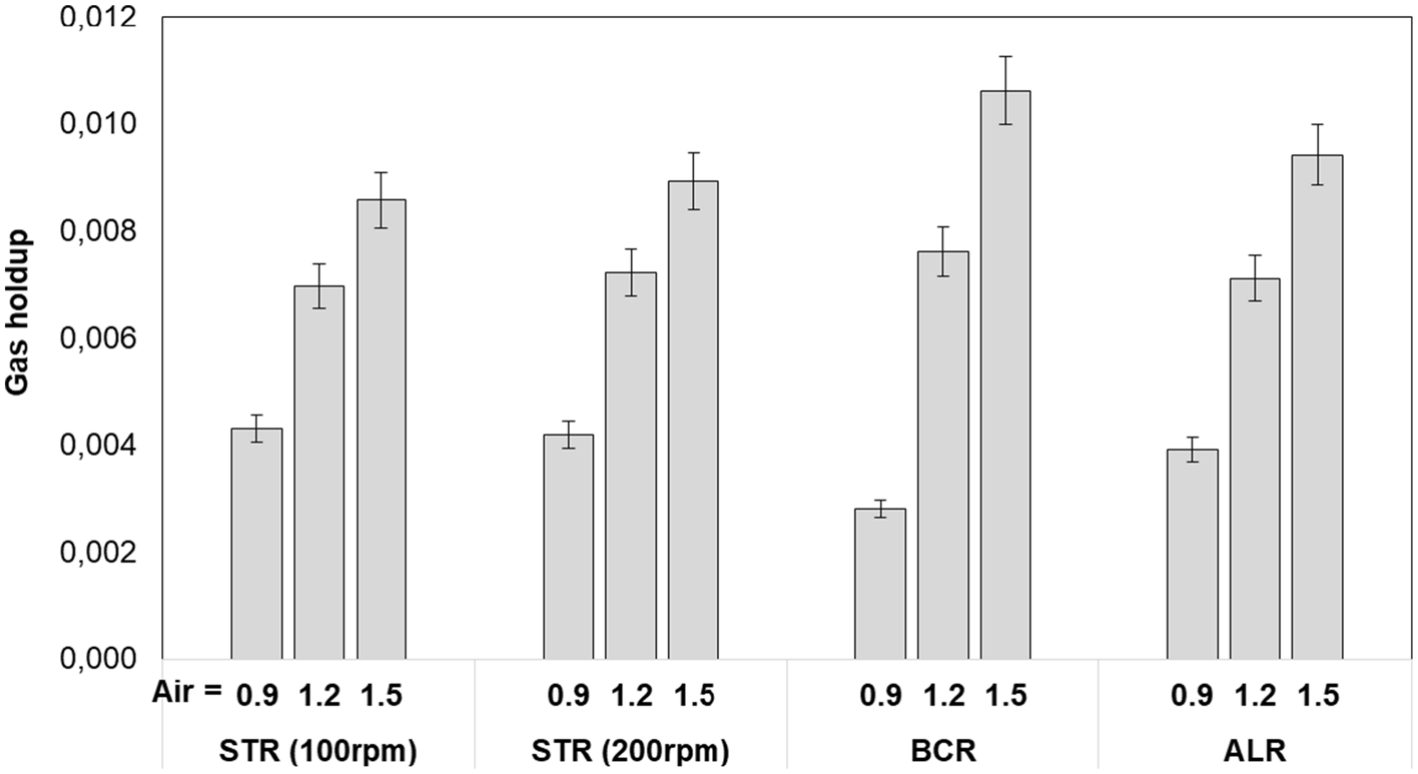
Effect of air flow rate (0.9, 1.2, and 1.5 L/min), and stirring speed of STR (100 and 200 rpm) on gas holdup of photobioreactors

Figure 4 represents the effect of air flow rate (0.9, 1.2, and 1.5 L/min) and the stirring speed (100 and 200 rpm, for STR only) on gas holdup of photobioreactors.

The importance of gas holdup in photo-bioreactors is multifold. The gas holdup determines the residence time of the gas in the liquid and, in combination with the bubble size, influences the gas–liquid interfacial area available for mass transfer [19, 42].

It can be deduced from Fig. 4 that the difference between gas holdups for different photobioreactors was insignificant. Tolulope (2019) who also compared ALR and BCR (H/D = 6.2) came to the same conclusion and reported that all the photobioreactor configurations exhibited a comparable gas hold-up range across the flow rates of 0.6−2.4 L/min [19].

There is a plethora of literature on the effect of airflow rate on gas holdup; in ALR and BCR type PBRs with high H/D ratio gas holdup increases linearly with air velocity, though the slope tends to decrease at high velocities [13, 16, 17, 21, 38, 43]. In our case, only three different gas velocities were tested, but in Fig. 4, it is evident that gas holdup increased linearly with increasing air velocity for all three photo-bioreactors. The linear increase of gas hold-up with the gas flow rate also indicated that the rising velocity of the bubbles (residence time of gas in the water) remained constant in the PBRs. Gas hold-up can also be affected by high pressure and the presence of particles in the liquid which can reduce bubble rising velocity, as reported in large (50–150L)-scale flat panel PBRs operating under tri-phasic conditions [13]. However those factors were also not effective in the present case due to small-scale PBRs and use of water as liquid phase. In BCR, linearity of gas holdup *vs* superficial gas velocity also indicate that the flow regime (homogeneous, transition, heterogeneous) did not change [17].

Increasing the stirring speed was also found to increase gas holdup very slightly (0.002 on average) in STR, possibly due to increased dispersion of bubbles. Pashaei et al. (2020) also reported a similar (~ 0.0025) increase in gas holdup as the stirrer speed increased from 100 to 200 rpm in a 3 L STR and suggested breaking of the large bubbles into small ones as the explanation of the increase in gas holdup [22]. Another study performed in a 13L STR with water also confirmed increase in gas holdup from 200 to 400 rpm stirrer speed due to better dispersion and retention of finer bubbles produced by the stirrer blades [24].

Gas holdup values reported in the literature cover a wide range, but were comparable to our data in similar setups and operating conditions. Notable ones are 0.02 in a 50L ALR for U_G_ = 0.0100 m/s [21], 0.3 in a 35L BCR for U_G_ = 0.3 m/s [43], 0.0045–0.0065 depending on top clearance in a 50L rectangular ALR for U_G_ = 0.0014 m/s [16], 12% in a 14L BCR for U_G_ = 0.0400 m/s [17], 0.025 in a 50 L flat panel photobioreactor for U_G_ = 0.0500 m/s [13], 8% in a 275L ALR for U_G_ = 0.0200 m/s [38], 0.018 in ALR for U_G_ = 0.0200 m/s [42], 0.025 in an 8L BCR for U_G_ = 0.0100 m/s [44], 0.09 in a 3L STR for 5L/min airflow rate [22],

[24], 1.7% − 7.8% in 15 mL ALR and BCR for airflow rate of 0.6 − 2.4 L/h [19].

### Power consumption

The bottleneck for the production of energy or commodities with microalgae is to develop more productive photobioreactor systems while reducing their cost [16].

Power consumption is a key parameter in the commercialization of the bioprocesses and bioreactors as it directly affects the operating cost. Power consumption in the photobioreactors is due to aeration, illumination, and stirring in STR.

Volumetric power consumption rates were calculated from Eq. 9 and 10 as 29.4, 39.2, 49.1 W/m^3^ in BCR and ALR for air flow rates of 0.9, 1.2, 1.5 L/min, respectively. For STR, additional power consumption (Eq. 11) of 0.65 W/m^3^ (for 100 rpm) or 1.30 W/m^3^ (for 200 rpm) due to stirring should be added to those values to find the combined power consumption.

STR had the highest power consumption of the three types of photobioreactors as expected, due to the presence of a mechanical stirrer.

In our photobioreactor setup, illumination required a constant consumption of 10W power per photobioreactor and was responsible for most of the power consumption, therefore any cost saving attempt should address illumination first. There is plenty of room for energy-saving efforts here; using less light, more energy efficient lamps, and 16 h light–8 h dark cycles instead of continuous illumination would all decrease energy consumption by illumination.

Additionally, using more energy efficient air pumps or stirring motors would also decrease total energy consumption.

### Liquid circulation time and liquid velocity

The liquid circulation in ALRs originates from the difference in bulk densities of the fluids in the riser and the down-comer. Liquid velocity affects turbulence, the fluid-reactor wall heat transfer coefficients, the gas–liquid mass transfer and the shear forces to which the microorganism are exposed [42].

In ALR, the liquid circulation time was determined to be 6.81 s, 6.59 s and 6.11 s for air flow rates of 0.9, 1.2, and 1.5 L/min (U_G_ of 0.57, 0.76, and 0.95 cm/s), respectively. Corresponding liquid velocities were found to be 10.86 cm/s, 11.23 cm/s, and 12.11 cm/s, respectively.

Liquid circulation velocities were not strong enough to entrain gas bubbles into the down-comer section and the gas phase was able to completely disengage from the liquid phase at the surface, as a result of which bubble-free regime was established in the down-comer section. Complete bubble circulation regime in which gas bubbles circulate with the liquid allows better phase contacting and mixing, therefore improves gas–liquid mass transfer, however require very high superficial air velocities and are not suitable for shear sensitive cultures.

The liquid velocity is controlled by the gas holdups in the riser and the down-comer, therefore is related to air flow rate and increases with increasing air velocity [16, 21, 42]. The results of this study support these previous findings and suggest a linear relationship between the gas flow rate and the liquid circulation time (or liquid velocity) (*R*^2^ = 0.98).

Our numerical results are also comparable to other published works, the liquid velocity was given to be between 5 and 13.6 cm/s in a 3L ALR [45], which is a size comparable to ours. In larger ALRs, higher liquid velocities were reported, 5–25 cm/s in a 50L rectangular ALR depending on air velocity and top clearance [16] and 19–29 cm/s in a 40L split cylinder ALR, where the authors also stated that liquid velocity was lower in draft tube ALR [21].

### Volumetric mass transfer coefficient (k_L_a)

Volumetric mass transfer coefficient (k_L_a) is the crucial characteristic of the photobioreactors and determines the capability of the photobioreactor to sustain optimum cell growth [16]. It is also often used to compare the efficiency of bioreactors, design and operation of mixing-sparging equipment and as an important scale-up factor [26, 42].

**Fig. 5:**
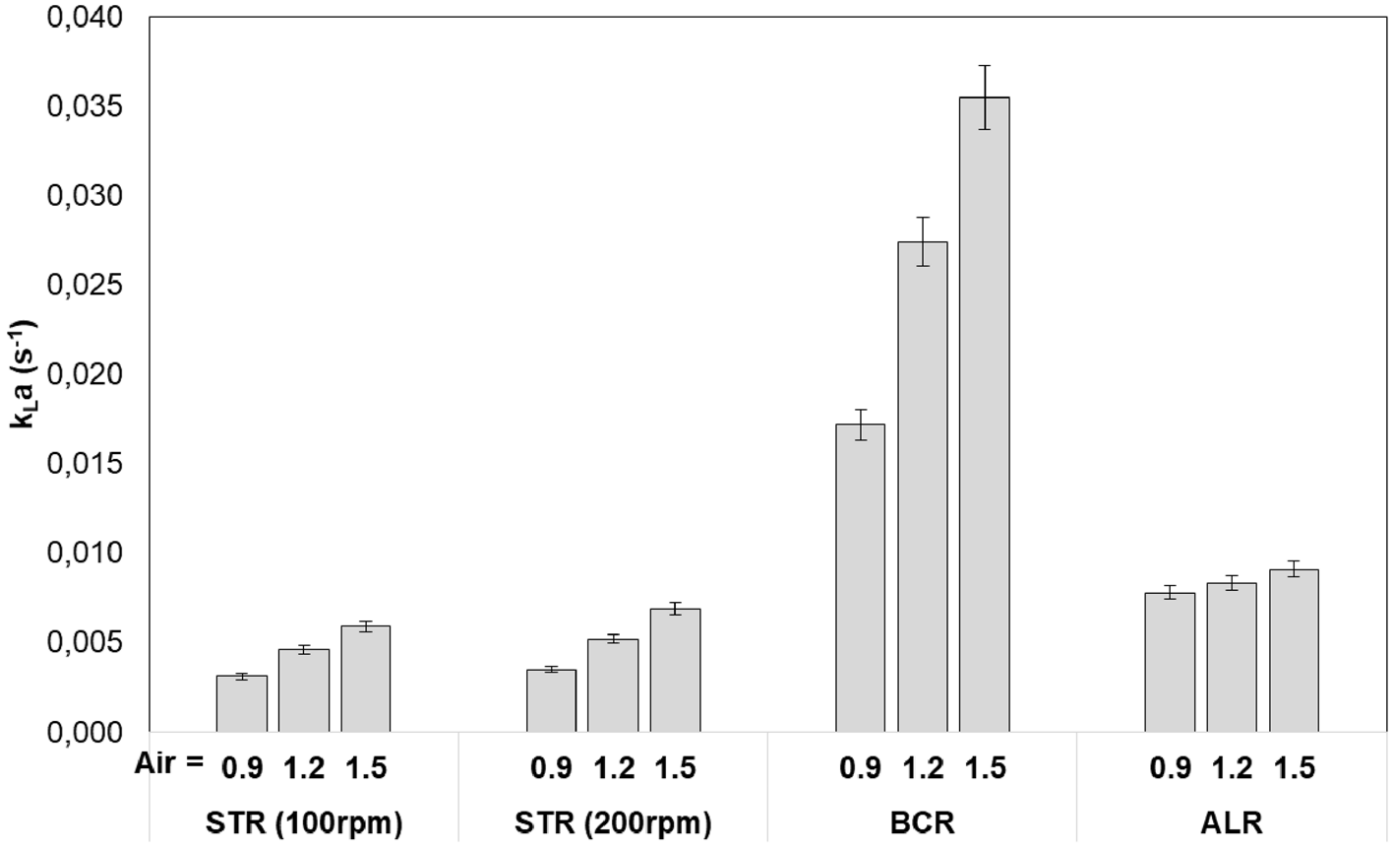
Effect of air flow rate and STR stirring speed on volumetric mass transfer coefficient (Air flow rates of 0.9, 1.2, and 1.5 L/min correspond to U_G_ of 0.30, 0.40, and 0.50 cm/s in STR and BCR, and to U_G_ of 0.57, 0.76, and 0.95 cm/s in ALR)

Figure 5 shows kLa of the photobioreactors in this study, for different air flow rates (0.9, 1.2, and 1.5 L/min), and stirring speeds of STR (100, 200 rpm).

The most significant finding is that the k_L_a value of BCR was more than five times than that of the STR and three times than that of the ALR. This difference can be attributed to the much smaller air bubbles in BCR, where a microporous sparger was used for aeration.

It is also evident from Fig. 5 that k_L_a depends on U_G_, it increased linearly as the U_G_ increased, in all cases.

In STR, stirring speed affected k_L_a values slightly, an increase from 100 to 200 rpm increased k_L_a by 15% on average.

Estimation of k_L_a from P/V for STR (Eq. 3) returns 0.0056, 0.0072, and 0.0088 s^−1^, for U_G_ of 0.3, 0.4, and 0.5 cm/s, respectively (stirring speed did not affect estimated kLa values significantly). Experimental values shown in Fig. 5 were between 0.0035 and 0.0069 s^−1^. The results are comparable, considering that the estimation have an error range of 20–40% and is suggested for 500 < P/V < 10,000 (W/m^3^) which is beyond our range.

For BCR, calculation of k_L_a from U_G_ based on proposed model (Eq. 4) yields 0.0100, 0.0127, and 0.0152 s^−1^ for U_G_ of 0.3, 0.4, and 0.5 cm/s, respectively. Corresponding experimental values shown in Fig. 5 were 0.0172, 0.0274, and 0.0355 s^−1^. Here, measured data were 70–133% higher than predicted values, which are more than error range of the proposed model (17%), additional error is possibly due to BCR size difference (*D* = 0.08 m & *H* = 0.4 m in this study, whereas correlation was proposed for *D* = 0.10–0.15 m & *H* = 2.5–4.4 m).

In ALR, kLa were predicted from Eq. 5 as 0.0053, 0.0074, and 0.0095 s^−1^ for air flow rates of 0.9, 1.2, and 1.5 L/min, respectively. Those were close to the measured experimental values of 0.0078, 0.0083, and 0.0091 s^−1^.

Our findings are consistent with those reached by others previously. It has been shown that k_L_a increases linearly with superficial gas velocity, however sometimes showing slight saturation kinetics or changing slopes at high velocities [13, 16, 17, 21, 43].

Miron et al. (2000) compared BCR to ALR and reported that k_L_a was reduced between 0 and 20% in ALR relative to BCR depending on the gas flow rate, and they explained the change by the reduced gas holdup [21]. Additionally, Tolulope (2019) found that volumetric mass transfer in BCR was greater than that in most shaken photobioreactors [19].

Kumar et al. (2012) also reported that k_L_a value in the same volume BCR was 28% higher than ALR [28].

The values of k_L_a reported previously vary greatly depending on U_g_ and the photobioreactor properties (i.e., scale, type) as illustrated in Table 1. The range of k_L_a for the 13 cases reported in the Table 1 is 0.0018–0.0755, with a mean of 0.0187 and a standard deviation of 0.0185 which is as high as the mean. However, for 1.5 L PBR volume and U_g_ of 0.0022 m/s, which is the closest setup to this study, k_L_a values of 0.0126 and 0.0161 were reported in ALR and BCR type PBRs, respectively, and those values are in the same range with this study (0.0078 and 0.0172 for ALR and BCR, respectively, at the lowest U_g_).

**Table 1:** Comparison of maximum k_L_a values reported in PBRs

Photobioreactor Type	Photobioreactor Size (L)	U_g_ (m/s)	k_L_a (s^−1^)	Reference
STR	2	0.0030–0.0050	0.0035−0.0069	This study
BCR	2	0.0030–0.0050	0.0172−0.0355	This study
ALR	2	0.0057–0.0095	0.0078−0.0091	This study
ALR and BCR	0.015	0.0008–0.0033	0.0017−0.0514	[19]
BCR	0.060	0.0030–0.0050	0.060–0.090	[47]
ALR and BCR	1.5	0.0022	0.012–0.016	[28]
BCR	9	0.010	0.024	[46]
BCR	14	0.040	0.0018	[17]
BCR	35	0.30	0.14	[43]
Rectangular ALR	50	0.0014	0.025	[16]
BCR and ALR	50	0.010	0.010	[21]
Flat panel	50	0.050	0.005	[13]
ALR	275	0.020	0.015	[38]

### Microalgae growth performance

*C. sorokiniana* growth performance of photobioreactors was compared under conditions that provided the highest k_L_a and the lowest mixing time; air flow rate was 1.5 L/min for all photobioreactors and stirring speed in STR was 200 rpm.

**Fig. 6:**
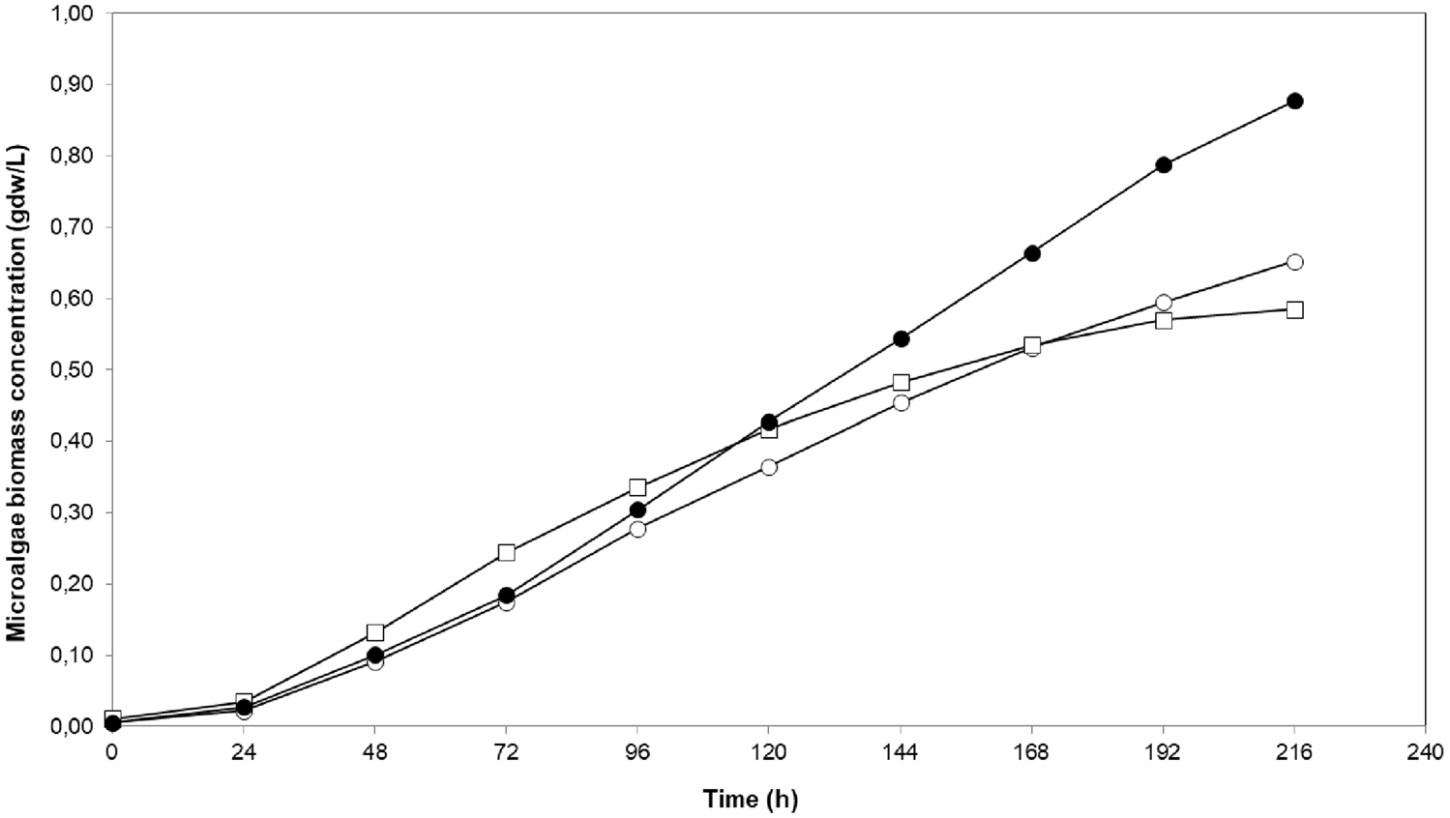
Comparative cell growth profile of *C. sorokiniana* in photobioreactors. (●): BCR, (□): STR, (○): ALR

Cell growth profiles in photobioreactors are given in Fig. 6. *C. sorokiniana* grew well in all three photobiroeactors indicating that all three photobioreactor types were suitable for microalgae cultivation. Exponential growth was observed for the first three days, after that linear growth dominated.

For STR, BCR, and ALR, maximum specific growth rates of microalgae were 0.053, 0.061, and 0.057 h^−1^, microalgae biomass productivities were 0.064, 0.097, and 0.072 gdw/L.day, and cell doubling times were 13.1, 11.4, and 12.2 h, respectively. BCR had a better performance compared to the other two photobioreactor types. Characterization data presented suggest that the volumetric mass transfer coefficient can be a feasible explanation for this difference.

In a comparable study, Tolulope (2019) also drew a similar conclusion, *C. sorokiniana* growth rate in BCR was 30% higher than the one in ALR (0.065 vs 0.085/h)[19].

Our results were compared to the literature in Table 2. Specific growth rates were generally on a par or higher than the ones reported in the literature, whereas the productivity values are lower.

**Table 2:** Comparison of performance for *C. sorokiniana* cultures

Photobioreactor type	Photobioreactor size	µ (/h)	Productivity (g/L.d)	Reference
STR	2 L	0.053	0.064	This study
BCR	2 L	0.061	0.097	This study
ALR	2 L	0.057	0.072	This study
Rectangular flask (shaken)	0.4 L	0.022	0.088	[48]
STR (550 rpm)	2 L	0.052	0.242	[49]
STR (300 rpm)	0.5 L	0.023	0.280	[50]
Ultra-thin	0.3 L	0.045	0.470	[51]
ALR	1.5 L	0.010	0.131	[28]
Flask (shaken)	0.6 L	0.018	0.043	[52]
BCR	0.3 L	0.035	0.143	[53]
ALR	0.015 L	0.065	0.223	[19]
BCR	0.015 L	0.084	0.320	[19]

It should be noted that we reported average productivity based on the full batch duration, and higher values are obtained during the exponential growth phase.

Temperature and pH directly affect the microbial growth and any deviation from optimal values may result in a significant response of the bioprocess [8].

In this study, even though pH and temperature were not actively controlled, they remained stable throughout the bioprocesses. During the runs, the temperatures of the photobioreactors were between 21 and 24 °C, the pH of the media was 7.8 initially, increased after the start of the runs to the alkali range within two days and stayed at around pH 10 until the end of the bioprocess (data not shown). There was no discernable difference between photobioreactors in terms of pH and temperature profiles, and those two parameters should not have affected specific growth rates and productivities.

The lipid and protein contents of the microalgae biomass were found to be 10 and 45%, respectively. It should be noted that high protein content found is due to the method used (Kjeldahl) which measures protein on the basis of total nitrogen content and does not distinguish protein-based nitrogen from non-protein nitrogen. Major fatty acids present in the microalgal biomass were palmitic acid (C16:1) 18%, oleic acid (C18:1n9c) 7%, linoleic acid (C18:2n6c) 24%, α-linolenic acid (C18:3n3) 17%. There was no detectable amounts of sugars (mono and disaccharides) or organic acids in the culture media.

## Conclusions and recommendations

In view of the findings discussed, the principal conclusions are as follows:

- The mixing times of all three photobioreactors were very low showing good homogenization in the vessels. BCR was marginally better than the other two. Mixing time depended on the air velocity for all three photobioreactors, and was slightly influenced by stirring speed in STR.
- Average bubble diameters in ALR and STR were similar. However, bubble size distribution in ALR was bimodal, whereas in STR, unimodal no-normal distribution was obtained.
- The difference between gas holdups for different photobioreactors was found to be insignificant. Gas holdup increased linearly with increasing air velocity for all three photobioreactors. Increasing stirring speed in STR was also found to increase gas holdup slightly.
- In ALR, a linear relationship between gas velocity and liquid circulation time (or liquid velocity) was shown.
- k_L_a value in BCR was much higher than in STR and ALR due to smaller air bubbles produced by the microsparger. It was also found that k_L_a increased linearly as the U_G_ and the stirring speed (in STR) increased.
- All three photobioreactors performed well in microalgae production and high specific growth rates were obtained. Under the conditions tested in this study, BCR had better microalgae growth performance compared to the other two photobioreactor types, possibly due to the significantly higher volumetric mass transfer.

The following issues would benefit from further research and can be recommended as follow-up studies:

- In-depth analysis of coalescence and breakup phenomena of gas bubbles depending on gas velocities for each PBR type can be useful to explain differences in microalgae growth performance observed in this study. Comparison of these PBRs to flat panel and tubular photobioreactors under the same conditions would be useful.
- Other height/diameter ratios can be investigated considering that H/D of 5 which was used in this study was higher than conventionally accepted range for STR, using a lower H/D ratio of 2 to 3 may improve mixing and increase microalgae growth performance.
- Repeating this study with on-line control of pH and monitoring the dissolved oxygen concentration on-line.

## Data Availability

The data that support the findings of this study are available from the corresponding author upon reasonable request.
